# “He’s Just a Wee Laddie”: The Relative Age Effect in Male Scottish Soccer

**DOI:** 10.3389/fpsyg.2021.633469

**Published:** 2021-01-28

**Authors:** James H. Dugdale, Allistair P. McRobert, Viswanath B. Unnithan

**Affiliations:** ^1^Physiology Exercise and Nutrition Research Group, Faculty of Health Sciences and Sport, University of Stirling, Stirling, United Kingdom; ^2^The Football Exchange, Liverpool John Moores University, Liverpool, United Kingdom; ^3^Research Institute for Sport and Exercise Sciences, Liverpool John Moores University, Liverpool, United Kingdom; ^4^Division of Sport and Exercise, School of Health and Life Sciences, University of the West of Scotland, Hamilton, United Kingdom

**Keywords:** RAE, adolescent, football, talent identification, development, selection, recruitment, Scotland

## Abstract

Significant structural, developmental, and financial constraints exist in Scottish soccer that may predicate a different approach to talent identification and development. To our knowledge, no published reports exist evaluating the prevalence of the relative age effect (RAE) in Scottish soccer players. Consequently, the aim of this study was to investigate the prevalence of the RAE among varied playing levels and ages of male Scottish youth soccer players. Birthdates of male youth players (*n* = 1,230) from U10 to U17 age groups and from playing levels: “Amateur” (*n* = 482), “Development” (*n* = 214), and “Performance” (*n* = 534), alongside a group of male Scottish senior professional players (*n* = 261) were recorded and categorized into quartiles (Q1 = January–March; Q2 = April–June; Q3 = July–September; and Q4 = October–December) and semesters (S1 = January–June and S2 = July–December) from the start of the selection year. Birthdates were analyzed for: (a) each playing level and (b) each age group irrespective of playing level. For the varied playing levels examined, an RAE was evident in “Development” and “Performance” playing levels only at youth level. When examining each age group, an RAE was observed in U12–U17 players only. While there was a slight asymmetry favoring Q1 born senior professional players, the RAE was not present within this group of our sample. Results from our study suggest that a bias in selecting individuals born earlier in the selection year may exist within male soccer academy structures, but not at amateur level. The asymmetry favoring chronologically older players at youth but not professional level questions the efficacy of this (un)conscious bias within male Scottish soccer players.

## Introduction

The relative age effect (RAE) is a well-established asymmetry in birthdate distribution favoring those born earlier in the selection year for a given sport (Cobley et al., 2009; Hancock et al., 2013). Scientists propose that RAE bias could occur due to individuals born earlier in the selection year growing and maturing ahead of their peers when grouped by chronological age (Wattie et al., 2008; Malina et al., 2017). It is also suggested that RAE bias could exist due to social factors, such as an earlier engagement in sporting activities or (un)conscious judgments by coaches influenced by physical factors related to growth and maturation (Hancock et al., 2013; Wattie et al., 2014). Although progress in understanding and communicating the RAE has been made over recent years, the “win now” emphasis encompassing youth sport remains, particularly within soccer (Andronikos et al., 2016; Reeves et al., 2018a; Hill et al., 2019; Lupo et al., 2019; Jackson and Comber, 2020). This demand for immediate success has resulted in a maturation-selection phenomenon, whereby chronologically older players are preferred due to their superior physical qualities (Lovell et al., 2015; Johnson et al., 2017; Hill et al., 2019). Thus, chronologically younger players may be overlooked or deselected during the developmental stages of talent development (Meylan et al., 2010; Helsen et al., 2012).

Differences in birthdate distributions in youth soccer are evidenced between academy players and the general population. For example, when comparing birthdate distributions of U8 English Premier League academy players to U8 regional grassroots players, a prevalent RAE was observed; with 57% of academy players born in the first quartile (Q1) of the selection year comparative to 30% for amateur grassroots players (Jackson and Comber, 2020). Similarly, Doncaster et al. (2020) observed that 53% of male soccer players from the FC Barcelona academy were born in Q1, compared to Q1 population values for Spain of 24%. An asymmetry in birthdate distribution favoring those born in either Q1 or the first semester (S1) of a selection year appears to be consistent within all age groups within professional soccer academies (Del Campo et al., 2010; Lovell et al., 2015), but not in amateur players (le Gall et al., 2010; Jackson and Comber, 2020). The magnitude of RAE in youth soccer tends to decrease with advancing chronological age, with authors observing the largest discrepancies in birth quartile distributions in comparatively young age groups (Helsen et al., 1998, 2000; Carling et al., 2009). Although not associated with physical growth and maturity factors at this stage, the RAE is proposed to be at its most influential within talent identification and (de)selection processes for pre-adolescent players (Lovell et al., 2015). Thereafter, biological maturation may further differentiate this already established asymmetry in birthdate distribution, cascading across the developmental process as exposure to systematic talent development grants them advanced coaching, competition, and facilities (Cobley et al., 2009; Lovell et al., 2015).

The prevalence of the RAE often continues into senior professional soccer succeeding any physical or biological advantages. For example, the RAE was evident in 9 out of 10 of the best leagues of the Union of European Football Associations (UEFA) during the 2016–2017 season (Yagüe et al., 2018). The RAE bias may transfer to professional levels due to the prevalence of birthdate asymmetry within the youth academy selection pool (Jimenez and Pain, 2008; Figueiredo et al., 2009). However, suggestions that the RAE diminishes as age increases and players transition to professional level, have been frequently reported (Mujika et al., 2009; González-Víllora et al., 2015; Skorski et al., 2016; Brustio et al., 2018; Gil et al., 2020). Considering the overrepresentation of chronologically older players at youth level, this reduction in prevalence of the RAE at senior professional levels challenges the efficacy of this (un)conscious bias.

It has been suggested that talented youth players not favored by the RAE bias (i.e., Q3/Q4 or S2 born) may be required to demonstrate superior technical and psychological skills to endure (de)selection during academy soccer (Cumming et al., 2018a; Patel et al., 2019). Consequently, when approaching senior professional transition, these superior abilities elevate these chronologically younger players due to the diminishing physical and developmental advantages previously benefitting their chronologically older peers (Votteler and Höner, 2014; Cumming et al., 2018b). Differing approaches to talent identification and (de)selection have been observed in soccer, largely influenced by the varied philosophies and perceived competition demands of the nation or league observed (Carling et al., 2012; Unnithan et al., 2012; Reeves et al., 2018b). Therefore, although there is evidence that the RAE is a global bias within the sport of soccer, it is logical to assume that its prevalence may vary dependent on the nation, and the under-pinning soccer philosophy of the club or nation, and competitive level examined.

Established soccer nations are highly successful at international level and often possess large talent pools, substantial participation rates, considerable financial and logistical resources, and strong domestic competition (Bennett et al., 2018). Comparatively, emerging soccer nations may be contrasting in these observations. When examining talent identification in soccer, the extant literature has largely focused on established nations (Figueiredo et al., 2009; le Gall et al., 2010; Carling et al., 2012; Unnithan et al., 2012; Reeves et al., 2018b). As a consequence, established soccer nations have made marked progress in identifying and addressing confounding factors of talent identification in soccer, whereas emerging nations may be limited due to the aforementioned discrepancies (Bennett et al., 2018). These limitations may result in a different approach to talent identification and development being adopted, and highlight the requirement for further investigation on confounding factors of talent identification and (de)selection in emerging soccer nations.

Despite high participation rates, an established national federation, and societal similarities to the rest of the United Kingdom, Scotland’s soccer success at both club and international levels has been hindered, potentially due to financial and developmental constraints (Morrow, 2006; Adams et al., 2017). Furthermore, Scotland has only recently implemented a systematic performance strategy, providing a framework for talent identification and development of youth players (SFA, 2017). Acknowledging these constraints, approaches to talent identification and development within Scottish soccer may not be comparable to published reports from the rest of the United Kingdom and Europe, and more in line with those of emerging soccer nations (Bennett et al., 2018). To our knowledge, no published reports exist that examine the prevalence of RAE in Scottish soccer players, and only one study reports on the RAE for a smaller, emerging soccer nation (Finnegan et al., 2017).

Therefore, the aim of this study was to assess the prevalence of the RAE in male Scottish youth soccer players across all ages and playing standards within the Scottish Football Association (SFA) National Governing Body infrastructure, compared to a reference group of male Scottish professional players. We examine birthdate distributions across quartiles and semesters for: (a) varied playing levels at youth (amateur, development, and performance) and in Scottish senior professional players and (b) each age group at youth (U10–U17), irrespective of playing level. It was hypothesized that the prevalence of the RAE would increase in-line with the playing standard classifications, but would be less apparent for senior professional players compared to youth. It was also hypothesized that the RAE would be most prevalent in younger players within our sample, cascading but gradually decreasing as players become older and physical and biological advantages diminish. These findings have the potential to provide insights for other, smaller countries that may not be comparable to more established soccer nations captured by the extant RAE literature.

## Materials and Methods

### Participants and Procedures

Birthdates of 1,230 male youth soccer players aged 9–17 years were obtained in February, 2020. Players were categorized into age groups: U10 (*n* = 54); U11 (*n* = 140); U12 (*n* = 164); U13 (*n* = 176); U14 (*n* = 192); U15 (*n* = 206); U16 (*n* = 180); and U17 (*n* = 118), and playing levels: amateur (recreational players, *n* = 482); development (lower ranked professional academies, *n* = 214); and performance (junior-elite academies, *n* = 534) as specified by the SFA (SFA, 2017). Birthdates were also obtained for 261 Scottish professional soccer players aged 17–38 years *via* Wyscout (Chiavari, Italy) in February, 2020. Professional players had competed in either the English Premiership, English Championship, or Scottish Premiership in the 12 months prior to data collection. The study received institutional ethical approval from the local University ethics committee.

### Statistical Analyses

Birthdates for all players were categorized into the following relative age quartiles: Q1 = January–March; Q2 = April–June; Q3 = July–September; and Q4 = October–December, and semesters: S1 = January–June and S2 = July–December from the start of the selection year specified by the SFA, and were reported as frequencies and percentages (%). The Chi squared (*χ*^2^) test was used to assess differences between observed and expected birthdate distributions across quartiles for: (a) each playing level and (b) each age group, irrespective of playing level. Expected birthdates were obtained from the National Records of Scotland and reflected average population birthdate distributions 1981–2010, capturing population records for the birth years from the oldest to the youngest players within our sample. Population birthdate distributions were reported as 24.8, 24.9, 25.6, and 24.7% for Q1, Q2, Q3, and Q4, respectively. Odds ratios (ORs) and 95% confidence intervals (95% CI) were calculated to compare the birthdate distribution of a quartile (Q1, Q2, or Q3) or semester (S1) with a reference group, consisting of the relatively youngest players (Q4 or S2, respectively). ORs were considered significant if the 95% CI range did not include a value ≤1.0. Data were analyzed *via* SPSS Statistics Version 25.0 for Windows (IBM, Chicago, Illinois, United States). Where appropriate, the alpha level was set at *p* < 0.05.

## Results

The frequency and percentage distributions of players’ birth quartiles for each playing level are presented in Table 1. Within the “Amateur,” “Development,” and “Performance” groups, players born in Q1 represented the largest quartile distribution across all playing levels, with a progressive decline to Q4. OR analyses revealed that an RAE existed within “Development” and “Performance” player groups, but not within the “Amateur” player group. The Chi-squared test demonstrated significant deviations across birth quartiles for “Development” and “Performance” groups only. For “Professional” players, players born in Q1 represented the largest quartile distribution; however, a progressive decline from Q1 to Q4 was not observed for this group. OR analyses revealed that an RAE did not exist for Scottish “Professional” players, and the Chi-squared test was not significant for this group.

**Table 1:** Birth quartile distributions by playing level.

	Birthdate distribution (%)					Odds ratio (95% CI)				Chi-squared
Playing level	*n*	Q1	Q2	Q3	Q4	Q1 vs. Q4	Q2 vs. Q4	Q3 vs. Q4	S1 vs. S2	*χ*^2^
Amateur	482	152 (31.5)	132 (27.4)	114 (23.7)	84 (17.4)	1.8 (0.8–4.1)	1.6 (0.7–3.6)	1.4 (0.6–3.1)	1.4 (0.8–2.5)	4.4
Development	214	92 (43.0)	50 (23.4)	40 (18.7)	32 (15.0)	2.9 (1.3–6.5)	1.6 (0.7–3.7)	1.3 (0.5–3.0)	2.0 (1.1–3.5)	19.1^*^
Performance	534	216 (40.5)	160 (30.0)	98 (18.4)	60 (11.2)	3.6 (1.5–8.5)	2.7 (1.1–6.4)	1.6 (0.7–4.1)	2.4 (1.3–4.3)	20.4^*^
Professional	261	76 (29.1)	56 (21.5)	65 (24.9)	64 (24.5)	1.2 (0.6–2.6)	0.9 (0.4–2.0)	1.0 (0.5–2.2)	1.0 (0.6–1.8)	1.2

Q1, January–March; Q2, April–June; Q3, July–September; Q4, October–November.

* Significant at an alpha level of *p* < 0.05.

The frequency and percentage distributions of players’ birth quartiles for each age group are presented in Table 2. For U10 and U11 player groups, the largest quartile distributions were observed in Q4 (29.6%) and Q3 (32.9%), respectively. However, this did not reach statistical significance. Players born in Q1 represented the largest birth quartile distribution for U12–U17 player groups (35.4–49.2%). For U12–U17 player groups, a progressive decline from Q1 to Q4 was observed. OR analyses revealed that an RAE existed across U12–U17 player groups, however, was highest for U17 players. The Chi-squared test demonstrated significant deviations across birth quartiles for U12–U17 groups only.

**Table 2:** Birth quartile distributions by age group.

	Birthdate distribution (%)					Odds ratio (95% CI)				Chi-squared
Age group	*n*	Q1	Q2	Q3	Q4	Q1 vs. Q4	Q2 vs. Q4	Q3 vs. Q4	S1 vs. S2	*χ*^2^
U10	54	12 (22.2)	16 (26.6)	10 (18.5)	16 (29.6)	0.8 (0.3–1.6)	1.0 (0.5–2.1)	0.6 (0.3–1.4)	1.1 (0.6–1.9)	3.3
U11	140	24 (17.1)	32 (22.9)	46 (32.9)	38 (27.1)	0.6 (0.3–1.4)	0.8 (0.4–1.9)	1.2 (0.6–2.6)	0.7 (0.4–1.2)	4.9
U12	164	58 (35.4)	50 (30.5)	38 (23.2)	18 (11.0)	3.2 (1.3–7.7)	2.8 (1.2–6.7)	2.1 (0.9–5.2)	1.9 (1.1–3.4)	13.6^*^
U13	176	78 (44.3)	56 (31.8)	20 (11.4)	22 (12.5)	3.6 (1.5–8.2)	3.6 (1.1–6.0)	0.9 (0.3–2.4)	3.2 (1.7–5.8)	31.2^*^
U14	192	84 (43.8)	40 (20.8)	38 (19.8)	30 (15.6)	2.8 (1.3–6.2)	1.3 (0.6–3.2)	1.3 (0.5–3.0)	1.8 (1.0–3.2)	19.9^*^
U15	206	82 (39.8)	68 (33.0)	34 (16.5)	22 (10.7)	3.7 (1.6–8.9)	3.1 (1.3–7.5)	1.6 (0.6–4.0)	2.7 (1.5–4.8)	22.9^*^
U16	180	64 (35.6)	52 (28.9)	44 (24.4)	20 (11.1)	3.2 (1.3–7.7)	2.6 (1.1–6.3)	2.2 (0.9–5.4)	1.8 (1.0–3.2)	12.9^*^
U17	118	58 (49.2)	28 (23.7)	22 (18.6)	10 (8.5)	5.8 (2.3–14.5)	2.8 (1.1–7.3)	2.2 (0.8–5.9)	2.7 (1.5–4.9)	36.6^*^
Professional	261	76 (29.1)	56 (21.5)	65 (24.9)	64 (24.5)	1.2 (0.6–2.6)	0.9 (0.4–2.0)	1.0 (0.5–2.2)	1.0 (0.6–1.8)	1.2

Q1, January–March; Q2, April–June; Q3, July–September; Q4, October–November.

* Significant at an alpha level of *p* < 0.05.

## Discussion

Considering the financial and developmental constraints facing Scottish soccer, combined with a paucity of published reports for smaller and emerging soccer nations, we explored the prevalence of the RAE among varied playing levels and ages of male Scottish soccer players. When examining the influence of playing level on the prevalence of the RAE in our sample, our results indicate that the RAE was evident in “Development” and “Performance” groups only. We also observed that the RAE was only prevalent in U12–U17 players, with no apparent RAE for U10–U11 players in our sample. Finally, while there was a slight asymmetry favoring Q1 born professional players, the RAE was not present within this group of our sample.

The finding that an RAE was particularly pronounced in youth academy soccer players (“Development” and “Performance”), compared to recreational players (“Amateur”), confirms our hypothesis and is consistent with previous reports (Del Campo et al., 2010; Lovell et al., 2015; Hill et al., 2019). It is widely proposed that physical factors associated with growth and maturation may influence talent identification and (de)selection decisions within academy soccer (Vaeyens et al., 2005; Wattie et al., 2008; Hancock et al., 2013), leading to an overrepresentation of chronologically older players within these settings. Moreover, chronologically older players may acquire more practice time due to an earlier onset in sporting engagement and, therefore, may be more likely to be recruited due to advanced technical abilities (Hancock et al., 2013; Wattie et al., 2014). Published reports suggest that chronologically older players may be taller, heavier, and perform better at physical fitness tests compared to their younger counterparts (Figueiredo et al., 2009). In addition, physical factors may provide acute technical advantages for youth players during competition (Meylan et al., 2010). The absence of a prevalent RAE in the “Amateur” players within our study suggests that birthdate asymmetries may occur due to selection and competition pressures associated with academy soccer, which are alleviated at grassroots level (Wattie et al., 2008; Lupo et al., 2019; Jackson and Comber, 2020). Although youth academies aim to promote developmental strategies to maximize chances of youth-professional transition, an overreliance on success and performance in the short-term has likely influenced decision-making within this sample (Andronikos et al., 2016). Our data provide further support to the premise that chronologically older players are overrepresented within male academy soccer. We also suggest that (un)conscious bias may be influencing (de)selection processes within our sample similar to previous observations from established soccer nations.

We also hypothesized that the RAE would be most prevalent in younger players and gradually decrease as players become older and physical and biological advantages diminish. Although our data demonstrate an asymmetry in birthdate distribution during the adolescent years, the prevalence of an RAE did not decrease with age as anticipated. It is proposed that physical differences, influenced by growth and maturation, may be most pronounced during early- and mid-adolescence (Malina et al., 2017). Therefore, the maturation-selection phenomenon may explain the prevalence of an RAE within players aged ~12–15 years within our sample. The observation that a significant asymmetry in birthdate distribution persisted until U17, however, suggests that selection bias continues to favor chronologically older players despite diminishing physical advantages. This is indicative of the “cascade effect,” suggesting a continuation of bias favoring chronologically older players during later years due to talent identification and (de)selection approaches during adolescence (Mujika et al., 2009; Lovell et al., 2015). Furthermore, this observation could be due to additional performance-related factors, such as technical ability or perceptual-cognitive skills, that may have been fostered during early recruitment and prolonged exposure to systematic talent development due to a birthdate advantage (Hancock et al., 2013; Wattie et al., 2014; Doncaster et al., 2020). Finally, we observed no RAE for U10–U11 players within our sample. This finding also supports the maturation-selection phenomenon, as evidence suggests that until the onset of adolescence, minimal differences in physical factors are evident between chronologically older and chronologically younger soccer players (Malina et al., 2010, 2012). We suggest that, comparative to previous reports, the asymmetry in birthdate distributions observed in our sample may be due to physical and biological factors influencing both (de)selection and participation from the onset of adolescence and cascading through to later years of youth soccer. However, our findings also support the notion that the RAE may extend beyond biological and physical advantages in sport, and that consideration to a wider array of potential factors contributing to the prevalence of the RAE should be prioritized (Hancock et al., 2013; Wattie et al., 2014; Doncaster et al., 2020).

Lastly, an RAE was not observed in our sample of male Scottish senior professional soccer players. This suggests that although the selection of chronologically older players seems to occur at youth levels, this approach may not translate to professional levels, thus questioning the efficacy of this (un)conscious bias. Although prevalence of an RAE have been previously reported for senior professional soccer players from established soccer nations (Helsen et al., 2012; Padrón-Cabo et al., 2016; Yagüe et al., 2018), these findings are ambiguous in nature and have been suggested to be dependent on the varied philosophies held by coaches or clubs, or by the perceived competition demands of the nation or league observed (Carling et al., 2012; Unnithan et al., 2012; Reeves et al., 2018b). Furthermore, the magnitude of birthdate asymmetries appears to reduce in established soccer nations between youth and professional levels (Mujika et al., 2009; González-Víllora et al., 2015; Skorski et al., 2016; Brustio et al., 2018; Lupo et al., 2019; Gil et al., 2020). Despite observing marked differences in RAE prevalence between academy and senior professional soccer players, these authors all report significant birthdate asymmetries at professional levels. Finally, considering that we observed the largest asymmetry in birthdate distribution for our U17 players, the age group before professional transition, it is possible that an RAE was prevalent in the early career phase of our professional players but reduced as players were (de)selected and progressed during their playing career, as previously observed (Lupo et al., 2019).

While we observed a slight asymmetry favoring Q1 born professional players, the RAE was not present within this group of our sample. This discrepancy between RAE prevalence in youth and professional players in our sample may support the notion that, over recent years, established soccer nations have made marked progress in identifying and addressing confounding factors of talent identification in soccer, whereas emerging and developing nations (which may be comparable to Scotland) may be limited due to financial, logistical, and competition limitations (Bennett et al., 2018). Furthermore, Scotland only recently implemented “Project Brave,” a systematic performance strategy for youth academies, comparable to the English Premier League’s Elite Player Performance Plan (The English Premier League, 2011), aligned to their philosophy and processes regarding long-term athlete development (SFA, 2017). Considering we observed a consistent RAE bias within our sample of Scottish academy soccer players, but not in Scottish senior professional players, could suggest that factors associated with birthdate advantages may have strongly influenced talent identification and (de)selection processes within Scotland during our data collection and prior to the inception of the SFA “Project Brave” Performance strategy in 2017. Resultant of this observation, we reiterate that acute physical advantages associated with birthdate advantages may not be representative of successful youth-professional transition, and their importance should be reconsidered during talent identification and talent development strategies.

This study is not without its limitations. Although we provide a comprehensive overview of birthdate distributions across multiple playing levels within organizational structures of male Scottish youth soccer, alongside a sample of male Scottish professional players, our sample is comparatively small considering previous examinations of RAE bias in soccer. However, we offer an investigation into a soccer nation that may not be comparable to the extant body of literature that focuses on established soccer nations, and suggest that this is considered in light of this limitation. Secondly, the absence of anthropometric and performance data is a further limitation of our study. As a consequence, our assumptions on the advantages associated with RAE biases, within our sample, are proposed upon their associations with chronologically older players rather than supported by original data. To progress this avenue of research further, future research should investigate talent identification and development structures in emerging and developing soccer nations, such as Scotland. Understanding these underlying mechanisms will allow progress to be made in reducing this bias and other confounding factors of (de)selection from youth soccer, and contribute toward a more systematic approach to talent identification and development.

## Conclusion

Results from our study suggest that a bias in selecting individuals born earlier within the selection year exists within male Scottish academy soccer, but not at amateur or professional levels. This bias did not diminish as age increased within our sample of Scottish youth soccer players, and was not present in U10–U11 age groups. Finally, when examining each age group within each playing level, we observed a relatively consistent RAE within our sample. Considering our findings, we suggest that the RAE may be a relatively consistent issue throughout all playing levels and ages of male Scottish youth soccer. As previously reported, birthdate asymmetries are particularly pronounced within youth academy settings, and that physical and social factors related to the RAE are likely influencing decisions around talent identification development in Scottish soccer. We encourage coaches and recruiters to acknowledge the prevalence of the RAE in Scottish soccer and draw attention to the potential limitations and exclusive nature of chronological age banding in sport.

## Data Availability Statement

The original contributions presented in the study are included in the article/supplementary material, further inquiries can be directed to the corresponding author.

## Ethics Statement

The studies involving human participants were reviewed and approved by the University of Stirling cross-faculty ethics committee. Written informed consent to participate in this study was provided by the participants’ legal guardian/next of kin.

## Author Contributions

JD, AM, and VU contributed to conception and design of the study. JD performed the data collection and wrote the first draft of the paper. JD and VU performed the data analysis. All authors contributed to manuscript revision and read and approved the submitted version.

### Conflict of Interest

The authors declare that the research was conducted in the absence of any commercial or financial relationships that could be construed as a potential conflict of interest.
